# Real-Time Sharing and Expression of Migraine Headache Suffering on Twitter: A Cross-Sectional Infodemiology Study

**DOI:** 10.2196/jmir.3265

**Published:** 2014-04-03

**Authors:** Thiago D Nascimento, Marcos F DosSantos, Theodora Danciu, Misty DeBoer, Hendrik van Holsbeeck, Sarah R Lucas, Christine Aiello, Leen Khatib, MaryCatherine A Bender, UMSoD (Under)Graduate Class Of 2014, Jon-Kar Zubieta, Alexandre F DaSilva

**Affiliations:** ^1^Headache and Orofacial Pain Effort (HOPE), Biologic and Materials Sciences DepartmentSchool of DentistryUniversity of MichiganAnn Arbor, MIUnited States; ^2^Department of Periodontics and Oral MedicineSchool of DentistryUniversity of MichiganAnn Arbor, MIUnited States; ^3^University of Michigan School of Dentistry (UMSoD)see Acknowledgements for collaborators; ^4^Translational Neuroimaging Laboratory, Molecular and Behavioral Neuroscience Institute (MBNI)University of MichiganAnn Arbor, MIUnited States; ^5^Michigan Center for Oral Health Research (MCOHR)School of DentistryUniversity of MichiganAnn Arbor, MIUnited States

**Keywords:** migraine, headache, epidemiology, social media, Twitter

## Abstract

**Background:**

Although population studies have greatly improved our understanding of migraine, they have relied on retrospective self-reports that are subject to memory error and experimenter-induced bias. Furthermore, these studies also lack specifics from the actual time that attacks were occurring, and how patients express and share their ongoing suffering.

**Objective:**

As technology and language constantly evolve, so does the way we share our suffering. We sought to evaluate the infodemiology of self-reported migraine headache suffering on Twitter.

**Methods:**

Trained observers in an academic setting categorized the meaning of every single “migraine” tweet posted during seven consecutive days. The main outcome measures were prevalence, life-style impact, linguistic, and timeline of actual self-reported migraine headache suffering on Twitter.

**Results:**

From a total of 21,741 migraine tweets collected, only 64.52% (14,028/21,741 collected tweets) were from users reporting their migraine headache attacks in real-time. The remainder of the posts were commercial, re-tweets, general discussion or third person’s migraine, and metaphor. The gender distribution available for the actual migraine posts was 73.47% female (10,306/14,028), 17.40% males (2441/14,028), and 0.01% transgendered (2/14,028). The personal impact of migraine headache was immediate on mood (43.91%, 6159/14,028), productivity at work (3.46%, 486/14,028), social life (3.45%, 484/14,028), and school (2.78%, 390/14,028). The most common migraine descriptor was “Worst” (14.59%, 201/1378) and profanity, the “F-word” (5.3%, 73/1378). The majority of postings occurred in the United States (58.28%, 3413/5856), peaking on weekdays at 10:00h and then gradually again at 22:00h; the weekend had a later morning peak.

**Conclusions:**

Twitter proved to be a powerful source of knowledge for migraine research. The data in this study overlap large-scale epidemiological studies, avoiding memory bias and experimenter-induced error. Furthermore, linguistics of ongoing migraine reports on social media proved to be highly heterogeneous and colloquial in our study, suggesting that current pain questionnaires should undergo constant reformulations to keep up with modernization in the expression of pain suffering in our society. In summary, this study reveals the modern characteristics and broad impact of migraine headache suffering on patients’ lives as it is spontaneously shared via social media.

## Introduction

Migraine affects approximately 12% of adults in the Western world [1]. In the United States, the prevalence of migraine is approximately 18% in women and 6% in men [2-4]. About 90% of migraineurs have moderate to severe pain during the attacks, 75% have reduced ability to function, and 30% require bed rest [4-6]. Although population studies have greatly improved our understanding of migraine, they have relied on retrospective self-reports that are subject to memory error and experimenter-induced bias. Furthermore, these studies also lack specifics from the actual time that attacks were occurring, and how patients express and share their ongoing suffering. Investigators have alluded to these limitations [7-11]; up until now, there have been no practical means to evaluate these observations in a geographically diverse population.

Infodemiology is a branch of science that deals with the occurrence, distribution, and analysis of electronic information that is used to inform the public of disease patterns and discourse, and of their relationship to the health status of a population. A key feature of infodemiology is the potential to collect and analyze data in near real time [12]. In this study, we explored the use of social media to evaluate migraine experience using Twitter, an online micro-blogging system. Twitter [13] allows registered users to post short text-based announcements known as “tweets”, consisting of a maximum of 140 characters, to an online public and accessible database. Tweets are instant, time-stamped, and self-reported communication from hundreds of millions of people worldwide. Tweets are usually built based on spontaneous reports with a natural self-expression, which makes social media a unique and innovative way to understand how communication and sharing of pain distress evolves. Twitter has been used as a key resource for public health surveillance, such as monitoring prescription drug abuse [14], smoking [15], and dietary behavior [16]. In addition, recent studies demonstrate that data retrieved from Twitter may be used to track dental pain [17], migraine [18], and to assess individual mood changes [19] and happiness [20], suggesting that this tool has the potential for describing universal human behaviors and patterns including emotional, social, and others [20,21]. The linguistics of suffering, as a broad context, is constantly modulated by factors such as social, cultural, and advances in technology. Additionally, the use of instant data avoids bias associated with retrospective reports, increasing accuracy and sensitivity of pain impact [11]. Nevertheless, analyses based on instant searching tools available through the Internet for social media may frequently lead to deceptive measurements due to the diversity of postings that are not all directly related to patient’s suffering as a result of migraine; for example, some tweets are associated with drug advertisement, the metaphoric use of the word “migraine”, and so forth [18]. To avoid these confounding factors and to estimate the instant impact of actual self-reported migraine headache suffering on the World Wide Web using Twitter, we analyzed the meaning and pattern of every single tweet message with the word “migraine” posted during an entire seven-day period.

Major aims of this study included using social media to assess migraine headache impact in real time to avoid memory bias, and to identify a set of current suffering descriptors that were not prompted by an experimenter. We report that Twitter, used as an instrument for infodemiology [12], is a rich source of information for migraine research with significant overlap of data from previously published large-scale epidemiological studies and has the potential to generate contemporary and clinically relevant results.

## Methods

### Study Design

A continuous cross-sectional sample of 21,741 tweets was collected between Saturday, April 30 (12:00:00 am) and Friday, May 6, 2011 (11:59:59 pm). According to the official Twitter website, a relative number of 1 billion tweets were posted weekly during that season, with an average of 200 million tweets per day from a total of 100 million active users [22,23]. In 2013, there were 200 million active users, tweeting an average of 400 million tweets per day [24]. The timeframe studied was randomly selected and it included seven consecutive days of uninterrupted posted messages (from Saturday to the following Friday). Hence, assumptions can only be drawn for this particular population that use this tool in social media. The data collected included only free, public Twitter user specific account information, which did not require any log-in data to be obtained.

### Ethics Statement

This study was certified as exempt from human subjects review by the University of Michigan Committee on Human Research (reference No. HUM00054476).

### Data Collection and Analysis

During the specified seven consecutive days, two investigators alternated in eight-hour shifts compiling all messages posted with the word “migraine” in the Twitter public search engine [25]. All the obtained results were then saved onto a main database. Then, in a systematic manner, three pain specialists oriented and supervised 54 undergraduates, four graduate dental students, and six research assistants on the reading, interpretation, and classification of the tweets into nine categories described below. Calibration lectures and sessions were performed with real-time samples from the Twitter webpage. Furthermore, students were divided into groups under the supervision of elected laboratory members, who answered individual questions during personal meetings and via email in case of uncertainty in the classification of a particular tweet. Participation in this project was only offered following formal lectures on primary headaches, especially migraine, and their classification guidelines based on the International Headache Society [26].

Subsequently, a coding system was used for in-depth interpretation and categorization of each tweet. The categories were: “migraine headache” (a user self-reporting having an actual migraine headache attack), “commercial” (advertising treatments or drugs), “metaphor” (the term migraine was used metaphorically), “not related” (the term migraine does not describe an actual physical experience of headache), “re-tweet” (a re-post of a previous tweet), “third person’s” migraine headache (information is related to another person’s migraine headache attack), general “discussion” (general discussion on migraine), “blanks” (missing data), and “inconclusive” (when not possible to identify the meaning of the word migraine in the tweet). In addition, when available, information about the self-reported migraine headache impact on the users’ sleep, work, social, school, mood, or debilitation was compiled using the same methods described earlier in the text. The following free and public information was also extracted: profile name, gender, and geographic location. All the acquired tweets were automatically translated to English by the Twitter website via Google Translate; however, only tweets originally written in English were used for linguistic analysis to avoid any translation bias.

We analyzed the time/date of occurrence of all the global self-reported migraine headache messages posted on Twitter using Greenwich Mean Time (GMT) for representation of the user time zone, ranging from 0h to 23h. However, the actual geographic location is discretionary information for the user and not always provided. Hence, to achieve a more comprehensive understanding of such temporal behavior, we isolated and then reported times in the United States (the largest representative group) by converting the geographic location time to the appropriate standard time, when available. For instance, an eight o’clock posting from a user on the East Coast in the United States was computed together with a similar posting from another user at local eight o’clock on the West Coast. To make sure every US tweet was corrected to the standard time zone, the US Census Bureau database [27] was used for each self-reported location, based on state and/or counties. For the states that have dual time zones, the same census database was used to estimate which time zone is more prevalent in the state, and that was used as the standard time zone. In addition, since the data was collected during a season when the majority of the United States observes Daylight Savings Time (DST), the US tweets were re-corrected for the local time when applicable. Therefore, we were able to precisely evaluate the temporal pattern of the self-reported migraine headache attacks posted on social media. The temporal pattern was based on a 24-hour time and days of the week.

All the data was compiled in Microsoft Excel, which was used to calculate basic descriptive statistics. Frequencies were reported for each category that was collected.

## Results

### Classification of Tweets in Categories

In a systematic manner, three pain specialists oriented and supervised 54 undergraduate students, four graduate students, and six research assistants on the reading, interpretation, and classification of tweets into the criteria described in the Methods section. For each posting, the following was taken into consideration: semantics, users’ demographics, impact of the attacks, geographic location, and time pattern.

**Figure 1:**
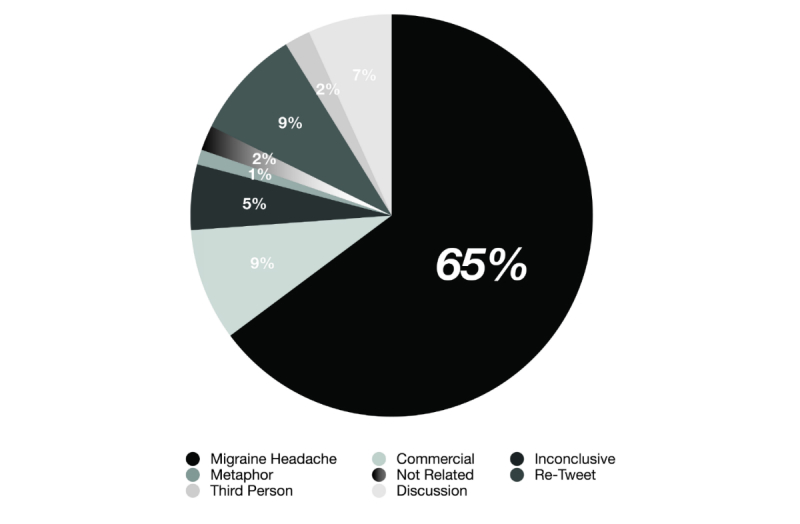
Classification of Tweets in Categories. Only 65% (14,028) of the 21,741 tweets were classified as self-reported migraine headache attacks.

Among the non-physical pain categories, advertising treatments or drugs (commercial) were the most prevalent, with 8.99% (1955 out of the 21,741 total tweets collected) prevalence. Re-tweets had a similar prevalence (8.85%, 1923/21,741 tweets), followed by general discussion (6.72%, 1462/21,741 tweets) or third person’s migraine (2.05%, 445/21,741 tweets), and metaphor (1.20%, 261/21,741 tweets). A total of 5.23% of the tweets were inconclusive (1137/21,741 tweets), 1.99% not at all related (434/21,741 tweets), and 0.44% were blanks (96/21,741 tweets). Only 64.52% of all the collected tweets (14,028/21,741) posted using the word “migraine” were an actual self-report of physical pain suffering and other migraine-related symptoms (Figure 1). Therefore, we used the 14,028 tweets (re-tweets and repeated tweets from the same account were not used) for our descriptive statistical analyses below that actually represent self-reported ongoing migraine headache (64.52% of a total of 21,741 tweets).

### Gender

**Figure 2:**
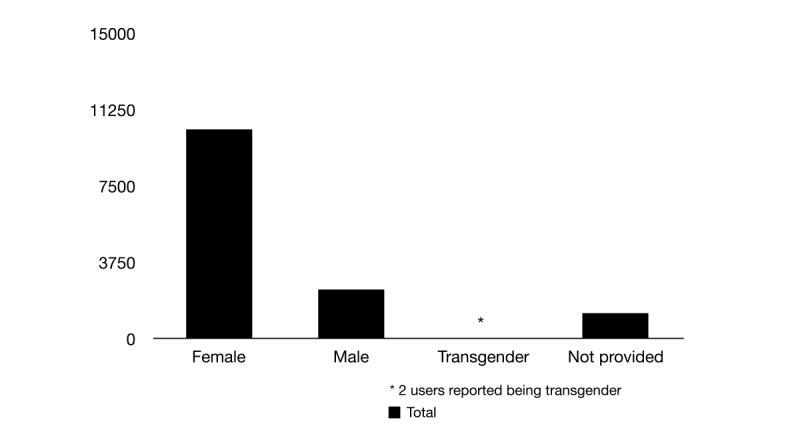
Gender distribution disclosed by users who reported their migraine headache attacks (n=14,028). 73.47% female (10,306 subjects), 17.40% male (2441 subjects), 0.01% (2 subjects) self-reported as transgender, and 9.12% was not provided (1279 subjects).

Based on the self-reported gender, it was found that 73.47% of the 14,028 migraine headache tweets were from females (10,306 tweets), 17.40% were from males (2441 tweets), 0.01% from transgender (2 tweets), and 9.12% was not provided (1279 tweets). The presence of transgendered in the assessment reflects a new trend in research studies, where the possibility of free self-expression leads to a more accurate gender representation of our modern society and cohort (Figure 2).

### Migraine Impact

**Figure 3:**
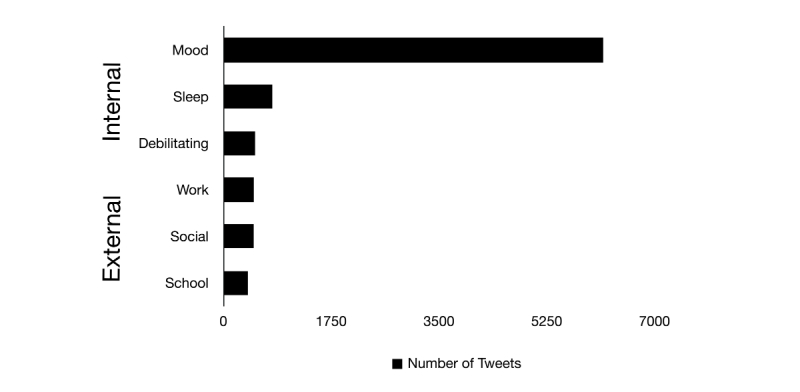
Impact and expression of migraine headache suffering (n=14,028). Personal impact (Internal) was predominantly on mood (43.91%, 6159 tweets). External impact was on productivity and absenteeism at work (3.46%, 486 tweets), social events (3.45%, 484 tweets), and school (2.78%, 390 tweets).

When possible, each interpretation of the tweets was also classified based on their migraine impact. The majority of the actual self-reported migraine headache posts generated an impact on patients’ internal status: personal impact, mostly “mood” with 43.91% (defined as any changes in the natural and emotional state of mind of the individual) (6159 tweets out of the 14,028 migraine tweets), followed by an impact on “sleep” with 5.61% (meaning difficulties falling and staying sleep) (787 tweets). Another percentage related to personal impact included “debilitating” with 3.61% (defined as a physically incapacitating migraine headache) (507 tweets). The external ongoing impact of the self-reported migraine attacks (productivity impact) similarly and instantly affected “work” productivity with 3.46% (impact on work productivity and/or absenteeism) (486 tweets), “social” life with 3.45% (denoting influence and/or absenteeism in current social activities) (484 tweets), and finally “school” with 2.78% (impact on school productivity and/or absenteeism) (390 tweets). Missing data in this category was 37.18% (5215 tweets) (Figure 3).

### Pain Descriptors

**Figure 4:**
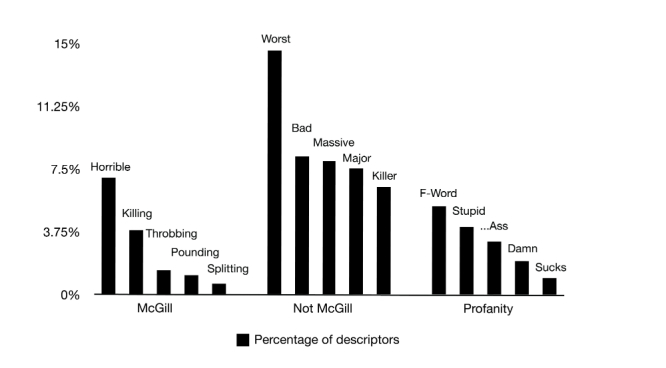
Most common pain descriptors used (n=1378). The most frequently used word from the McGill Pain Questionnaire (MPQ) was "horrible" (6.97%, 96 uses). Additional migraine headache adjectives (“Not McGill” words) included "worst" (14.59%, 201 uses) and profanity, the "F-word" (5.30%, 73 uses) being most frequently used.

Based on the physical tweets originally posted in English, we compared the adjectives used to describe a real ongoing migraine attack with the McGill Pain Questionnaire (MPQ) [28], one of the most widely used pain descriptor and rating questionnaires in research. In total, there were 242 descriptors used; however, there were only 45 English descriptors used a total of 1378 times. A prevalence of the word “horrible” was evident with 6.97% (used in 96 of the 1378 tweets with descriptors), followed by: “killing” (3.85%, used 53 times), “throbbing” (1.45%, 20 uses), “pounding” (1.16%, 16 uses), and “splitting” (0.65%, 9 uses). Conversely, subjects also expressed their migraine using words not included in MPQ. For classification purposes, we called these words “Not McGill”. The most frequently expressed words in this category included: “worst” (14.59%; 201/1378 uses), “bad” (8.27%, 114 uses), “massive” (7.98%, 110 uses), “major” (7.55%, 104 uses), and “killer” (6.46%, 89 uses). Interestingly, profanity was also highly used to describe the ongoing migraine attack suffering, with the “F-word” being the most frequent in that category (5.30%, 73 uses). Furthermore, “stupid” (4.06%, 56 uses), “…ass” (3.19%, 44 uses), “damn” (2.03%, 28 uses), and “sucks” (1.02%, 14 uses) were also used (Figure 4).

### Geographic Distribution

**Figure 5:**
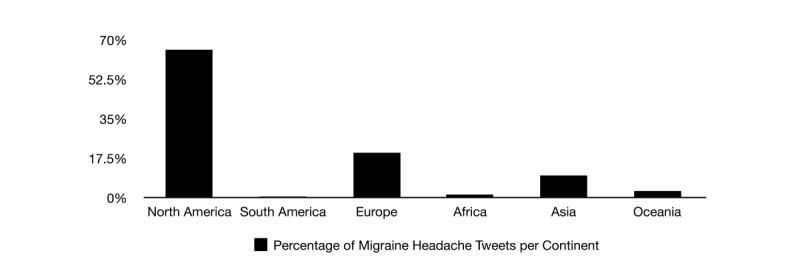
Percentage of migraine headache tweets by continent (n=5865). Most represented was North America with 65.57% (3840); United States alone represented 58.28% of overall data (3413 tweets).

The collected database is geographically diverse, since it was composed of real-time tweets from all around the world. To better visualize and understand the origin of the messages, we divided the posts based on self-reported geographic location. When in doubt about the precise location, Google Maps [29] was used to help precisely locate the region using the Global Positioning System (GPS), if the user made the geographical coordinates publicly available. The vast majority of the tweets came from North America (65.57%, 3840 out of the 5856 Twitter users who reported their location), followed by Europe (19.89%, 1165 tweets), Asia (9.80%, 574 tweets), Oceania (2.92%, 171 tweets), Africa (1.33%, 78 tweets), South America (0.48%, 28 tweets), and last, Antarctica with no tweets reported. The United States alone represented 58.28% of the data (3413/5856 tweets) (Figure 5).

### Temporal Pattern by Hour and Day of the Week

To have a better understanding of the online temporal behavior of sharing and expression of actual ongoing migraine headache suffering on the database, we organized each tweet based on the time and day when it was posted. As an initial step, we divided each global tweet by the day of the week it was posted. Our analysis demonstrated a higher global prevalence of self-reported migraine attack tweets on Tuesday (2559 of the 14,028 tweets) and Thursday (2155). It was followed by Wednesday (2074), Saturday (1933), Monday (1909), and Sunday (1752). A lower global prevalence of self-reported migraine headache tweets was observed on Friday (1646).

**Figure 6:**
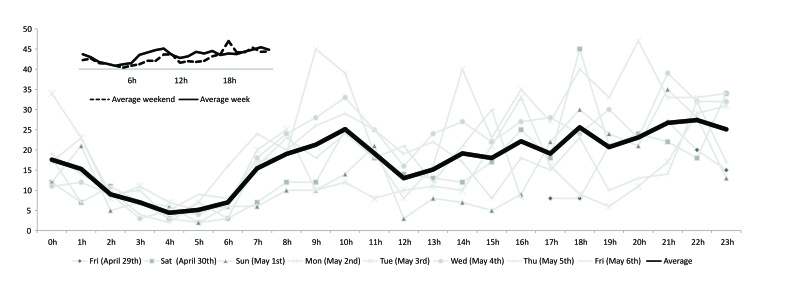
Temporal patterns of migraine headache tweets in the United States. Tweets were converted to local times and corrected for daylight savings time. The averaged flow of migraine tweets accumulated at 10h, persisted and gradually peaked later at night (22h). During Saturday and Sunday (dashed line in the top-left graph), the highest peak occurred at 18h.

In an effort to improve our temporal data interpretation, the posts were divided according to the reported GMT, since it is the traditional method used by the Twitter website. By plotting the information on a timeline graphic, it was possible to observe a peak of the global migraine headache-related tweets at 14:00 GMT on Monday. This valuable information could easily lead to misleading interpretations if not adjusted for the original standard time of each specific geographic region where the tweets were generated. Consequently, we selected the United States (since it had the majority of tweets) and converted each single tweet to their particular local time. The United States observes DST during the spring season in several regions, which could also lead to erroneous interpretation if not corrected for this particular time when appropriate, during this period of the year. Ultimately, 9:00h and 20:00h on Monday across the United States were the actual peaks of prevalence when most Americans were sharing on social media the occurrence of their migraine headache attacks (Figure 6). When all weekday tweets, from Monday to Friday, were averaged based on the timeline, this first morning peak of migraine headache attack postings shifted rightward to 10:00h, and then from midday, it gradually and steadily peaked again at 22:00h. The weekend days, Saturday and Sunday, had a similar two-peak pattern of postings, though with a later morning peak at 11:00h, and an earlier and higher night peak of tweets at 18:00h (Figure 6).

## Discussion

### Principal Findings

An unbiased evaluation of spontaneous sharing and expression of an ongoing migraine headache suffering is crucial for clinicians and researchers to understand the pattern of the disorder, and most importantly, the population under study. Here, we report the use of Twitter as a research tool to assess epidemiology and linguistics of migraine suffering in real-time in our modern society. Our results showed not only a significant overlap with other traditional epidemiologic studies, but also generated unique information about *who, what, how, where,* and *when* ongoing migraine headache suffering is shared on social media.

The methodology used here provided an effective, but laborious and time-consuming approach, to analyze reports of real-time migraine headache attacks on social media. This step was extremely important to avoid any sort of erroneous interpretation in our study. The use of current generalized algorithm search tools available on the Internet to effortlessly analyze altogether a sample of migraine tweets could inevitably lead to misleading conclusions, since they currently struggle to precisely exclude tweets that contain the word “migraine” used for commercial advertisement, general discussion, re-tweets, and metaphors [18]. In our database, only 64.52% of the migraine tweets (14,028) were an actual self-report of an ongoing migraine headache suffering. Therefore, all the analysis generated from this data was exclusively made using a sample of tweets from subjects reporting the occurrence of their own migraine attacks. Although we could not verify the accuracy of their diagnosis, in large computer-assisted telephone interview studies in the United States and abroad, individuals who call their headaches a migraine are about three times more likely to have a true migraine, based on the International Headache Society criteria [26], than those who are unaware of the type of the headaches they are suffering [30,31].

In this study, 73.47% of those who self-reported migraine headache were females (10,306 subjects of the 14,028 users who self-reported migraine) and 17.40% were males (2441 subjects). These results demonstrate and reinforce a higher prevalence of migraine headaches among females, which is consistent with population-based studies performed around the world [4,31]. Similar results have also been demonstrated in a recent study investigating the relative number of migraine searches and self-reports on Google and Twitter [18], respectively. Nonetheless, an in-depth evaluation of individual tweets was not provided in that particular study. The observed higher prevalence of migraine headaches in females could possibly be augmented by gender differences in expressing suffering or simply predilection of female users on social media. Nevertheless, real-time epidemiological studies that elaborate on point prevalence of pain/migraine and gender are lacking. Intriguingly, our study also shows that migraine headache suffering was prevalent in 0.01% (2 subjects) of Twitter users that freely self-described themselves as transgendered. Although minor in prevalence, the information for this gender group is not usually collected and investigated by current epidemiological studies, which highlights the importance of the inclusion of such gender identities in future research for better representation of migraine populations that fall outside the conventional female/male classification.

Migraine is a disabling form of primary headache [5], and the reports from patients on social media reflect what it impacts in life and society. Traditional (non-social media) epidemiological studies have emphasized the significant migraine-related impairment on productivity of routine and leisure activities [6,32]. However, the instant personal impact of migraine headache attacks freely reported in the tweets was predominantly on mood status (43.91%, 6159 of the 14,028 migraine headache tweets), which confirms the strong role of social media in affective expression [19] even when related to health issues. Second to mood status, the instant impact of migraine headache attacks was on sleep quality (dysfunctions to fall or stay asleep) on a minor level. This finding is consistent with previous studies that show a decrease in sleep quality of migraineurs [33,34]. Last, the external impact of the migraine headache attacks was immediate, as reported by migraine sufferers via tweets, and comparable on productivity and absenteeism at work, social events, and school (Figure 3). Since impact can be considered a qualitative variable, and possibly the most challenging category in this data analysis, all the students involved in this project received specific training and orientation. This was accomplished by calibration lectures based on real-time search and classification of tweets with the word migraine. All lectures in which this calibration was made were based on the classification guidelines of the International Headache Society [26]. In addition, each student was assigned a laboratory mentor in case of questions. All the training was performed prior to starting the classification, in order to ensure consistent categorization of the migraine impact.

What inevitably differentiates human from animal pain research is our ability to articulate the suffering experience and how we communicate it constantly evolves. In the case of migraine, the pulsating nature of the attacks has commonly been described in scientific literature as “throbbing”, and is even included in the International Headache Society criteria for migraine diagnosis [26]. Nonetheless, migraineurs freely defined their migraine headache attacks in multiple ways on Twitter. When restricted to the MPQ [28], a predetermined list of words and ratings widely used in medical contexts for measurement and assessment of pain, the most frequently used MPQ word in their tweets was “horrible”. It should be noted that “horrible” is used in the questionnaire to designate the pain intensity level and not pain quality. “Throbbing” was only the third MPQ word used, which was preceded by “killing”, an affective descriptive word. Additional migraine headache attack adjectives, categorized as “Not McGill” words in this study, were twice as prevalent as the MPQ words. “Worst” and “bad” were the leading migraine descriptors in this group, indicating mostly the severity differentiation by subjects of their ongoing attacks from the common ones. In addition, as the formality of doctor-patient communication is non-existent in Twitter, patients also felt understandably entitled to use profanity during actual suffering, with the “F-word” being the most frequently uttered to describe their migraine headache attacks. In summary, linguistics of ongoing migraine reports on social media proved to be highly heterogeneous and colloquial in our study, suggesting that current pain questionnaires should undergo constant reformulations to keep up with modernization in the expression of pain suffering in our society.

Real-time expression of migraine suffering occurs daily at the global level via social media. It is worth highlighting that it was at 14:00 GMT on the Monday that our planet had the highest flow of migraine headache attack postings on Twitter. The majority of those postings originated from North America, where the United States represented more than half of the total global stream. When each single tweet across the United States was converted to its particular local time, and corrected for DST during spring season when necessary, the largest point prevalence of migraine headache suffering was clustered on Monday with one peak in the morning at 9:00h and another one at 20:00h (Figure 6). As the 9:00h peak of tweets possibly reflected the onset of communication of migraine headache attacks in the early mornings [35], the second and gradual peak at night indicated that the duration of the migraine headache suffering, and its expression through social media, persisted and accumulated throughout the day. Curiously, general negative affect communications on social media also tend to rise throughout the day to a similar nighttime peak [19], and the emotional impact of migraine may potentiate the end of the working-day blues and vice-versa. In fact, when averaged with the other weekdays (Monday to Friday), the flow of migraine headache attack postings persisted and peaked even later at night. This changed during Saturday and Sunday, when people usually decompress from work-related stress and awaken later. Consequently, the initial morning peak of migraine headache attack postings during the weekend was further delayed; however, there was an earlier and high rise of ongoing migraine headache suffering postings at night, especially on Saturday. However, it is worth mentioning that the relatively short sample time may not provide the most accurate representation regarding the flow of migraine headache postings on social media.

### Conclusion

This study showed that the spontaneous flow of communication on Twitter reflects multiple patterns of human interaction for sharing the ongoing suffering experience, and proved to be a rich and instant resource of knowledge regarding the actual impact of migraine attacks in our modern society.

## Abbreviations

DST: Daylight Savings Time
GMT: Greenwich Mean Time
MPQ: McGill Pain Questionnaire

## References

[1] Rasmussen BK (1995). Epidemiology of migraine. Biomed Pharmacother.

[2] Lipton RB, Stewart WF, Simon D (1998). Medical consultation for migraine: results from the American Migraine Study. Headache.

[3] Lipton RB, Stewart WF, Diamond S, Diamond ML, Reed M (2001). Prevalence and burden of migraine in the United States: data from the American Migraine Study II. Headache.

[4] Lipton RB, Bigal ME, Diamond M, Freitag F, Reed ML, Stewart WF, AMPP Advisory Group (2007). Migraine prevalence, disease burden, and the need for preventive therapy. Neurology.

[5] Bigal ME, Serrano D, Reed M, Lipton RB (2008). Chronic migraine in the population: burden, diagnosis, and satisfaction with treatment. Neurology.

[6] Stewart WF, Lipton RB, Simon D (1996). Work-related disability: results from the American migraine study. Cephalalgia.

[7] Kikuchi H, Yoshiuchi K, Miyasaka N, Ohashi K, Yamamoto Y, Kumano H, Kuboki T, Akabayashi A (2006). Reliability of recalled self-report on headache intensity: investigation using ecological momentary assessment technique. Cephalalgia.

[8] Stone AA, Schwartz JE, Broderick JE, Shiffman SS (2005). Variability of momentary pain predicts recall of weekly pain: a consequence of the peak (or salience) memory heuristic. Pers Soc Psychol Bull.

[9] Erskine A, Morley S, Pearce S (1990). Memory for pain: a review. Pain.

[10] Stone AA, Broderick JE (2007). Real-time data collection for pain: appraisal and current status. Pain Med.

[11] Gendreau M, Hufford MR, Stone AA (2003). Measuring clinical pain in chronic widespread pain: selected methodological issues. Best Pract Res Clin Rheumatol.

[12] Eysenbach G (2009). Infodemiology and infoveillance: framework for an emerging set of public health informatics methods to analyze search, communication and publication behavior on the Internet. J Med Internet Res.

[13] Twitter Inc.

[14] Hanson CL, Cannon B, Burton S, Giraud-Carrier C (2013). An exploration of social circles and prescription drug abuse through Twitter. J Med Internet Res.

[15] Myslín M, Zhu SH, Chapman W, Conway M (2013). Using Twitter to examine smoking behavior and perceptions of emerging tobacco products. J Med Internet Res.

[16] Hingle M, Yoon D, Fowler J, Kobourov S, Schneider ML, Falk D, Burd R (2013). Collection and visualization of dietary behavior and reasons for eating using Twitter. J Med Internet Res.

[17] Heaivilin N, Gerbert B, Page JE, Gibbs JL (2011). Public health surveillance of dental pain via Twitter. J Dent Res.

[18] Linnman C, Maleki N, Becerra L, Borsook D (2013). Migraine tweets - what can online behavior tell us about disease?. Cephalalgia.

[19] Golder SA, Macy MW (2011). Diurnal and seasonal mood vary with work, sleep, and daylength across diverse cultures. Science.

[20] Dodds PS, Harris KD, Kloumann IM, Bliss CA, Danforth CM (2011). Temporal patterns of happiness and information in a global social network: hedonometrics and Twitter. PLoS One.

[21] Bell G, Hey T, Szalay A (2009). Computer science. Beyond the data deluge. Science.

[22] Twitter Inc.

[23] Twitter Inc.

[24] Twitter Inc.

[25] Twitter Inc.

[26] Headache Classification Subcommittee of the International Headache Society (2004). The International Classification of Headache Disorders: 2nd edition. Cephalalgia.

[27] US Department of Commerce US Census Bureau database.

[28] Melzack R (1975). The McGill Pain Questionnaire: major properties and scoring methods. Pain.

[29] Google Maps.

[30] Lipton RB, Stewart WF, Liberman JN (2002). Self-awareness of migraine: interpreting the labels that headache sufferers apply to their headaches. Neurology.

[31] Stovner Lj, Hagen K, Jensen R, Katsarava Z, Lipton R, Scher A, Steiner T, Zwart JA (2007). The global burden of headache: a documentation of headache prevalence and disability worldwide. Cephalalgia.

[32] Stang PE, Osterhaus JT (1993). Impact of migraine in the United States: data from the National Health Interview Survey. Headache.

[33] Seidel S, Hartl T, Weber M, Matterey S, Paul A, Riederer F, Gharabaghi M, Wöber-Bingöl C, Wöber C, PAMINA Study Group (2009). Quality of sleep, fatigue and daytime sleepiness in migraine - a controlled study. Cephalalgia.

[34] Engstrøm M, Hagen K, Bjørk MH, Stovner LJ, Gravdahl GB, Stjern M, Sand T (2013). Sleep quality, arousal and pain thresholds in migraineurs: a blinded controlled polysomnographic study. J Headache Pain.

[35] Fox AW, Davis RL (1998). Migraine chronobiology. Headache.

